# Integrating Pre-test Probability and Point-of-Care Ultrasound (POCUS) in the Emergency Department (ED) Diagnosis of Small Bowel Obstruction (SBO)

**DOI:** 10.7759/cureus.56397

**Published:** 2024-03-18

**Authors:** Katarzyna Krol, Yi-Ru Chen, Melva O Morales Sierra, Rida Nasir, Naya Mahabir, Lisa Iyeke, Lindsay Jordan, Trupti Shah, Kevin Burke, Matthew Friedman, Daniel Dexeus, Athena Mihailos, Mark Richman, Joshua Guttman

**Affiliations:** 1 Emergency Medicine, New York University Grossman School of Medicine, New York, USA; 2 Emergency Medicine, Northwell Health, New Hyde Park, USA; 3 Emergency Medicine, Long Island Jewish Medical Center, Northwell Health, New Hyde Park, USA; 4 Internal Medicine, Icahn School of Medicine at Mount Sinai, New York, USA; 5 Emergency Medicine, Emory University School of Medicine, Atlanta, USA

**Keywords:** ed, emergency department, nns, number needed to scan, emergency medicine, point-of-care ultrasound, pocus, sbo, small bowel obstruction

## Abstract

Introduction

A computed tomography (CT) scan and point-of-care ultrasound (POCUS) are commonly employed for diagnosing small bowel obstructions (SBOs). Prior studies demonstrated that POCUS has 90-95% sensitivity and specificity compared with CT scanning, which is the gold standard. Unlike other imaging modalities (in which the ordering and performing clinician are not the same), POCUS-performing/interpreting sonologists must recognize the risk of confirmation bias in the POCUS application. Per Bayesian analysis, the likelihood of a diagnosis being true following a diagnostic test is based on the ordering clinician’s pre-test probability and the test characteristics (sensitivity and specificity, from which positive and negative likelihood ratios can be calculated). Consequently, establishing pre-test probability is important in informing downstream diagnostic or therapeutic interventions, as pre-test probability influences post-test odds. Little research has been done on the role of POCUS sonologist’s pre-test probability and actual POCUS results regarding SBO. This study assessed the role of POCUS, integrating pre-test probability and POCUS results to determine post-test odds.

Methods

One hundred six patients were recruited on a convenience basis and underwent POCUS and CT between April 2017 and December 2022. All sonographers were credentialed in POCUS. POCUS sonologists’ pre-test probabilities and POCUS and CT results were captured, which were compared. Sensitivity, specificity, LR+, and LR− were calculated, and correlations were made between pre-test probability and POCUS and CT results.

Results

POCUS exhibited a sensitivity of 92% and specificity of 90%, with a corresponding positive likelihood ratio (LR+) of 9.3 and a negative likelihood ratio (LR−) of 0.09 for diagnosing SBO. Among patients with a high pre-test probability of SBO, a negative ultrasound yielded post-test odds of 0.4%, whereas a positive POCUS yielded post-test odds of 39.6%. Among patients with a low pre-test probability, a negative POCUS resulted in post-test odds of 0%, while a positive POCUS led to post-test odds of 2.1%, yielding a number needed to scan (NNS) of ~50 to identify a patient with an SBO on CT.

Conclusion

This study confirmed POCUS’s sensitivity and specificity of ~90-95% and a corresponding LR+ of 9.2 and LR− of 0.9. Pre-test probability substantially affected post-test odds. Patients with a high pre-test probability and a positive POCUS had post-test odds of 39.6 and should have a confirmatory CT, while those with a negative POCUS have very low post-test odds and very likely will not benefit from CT. Patients with low pre-test probability and a positive POCUS have post-test odds of 2.1%, similar to the Wells Score and HEART score; such patients may not benefit from a CT, though clinicians should use their judgment/discretion. Patients with a low pre-test probability and a negative POCUS have post-test odds of 0% and should not have a CT. Among low pre-test probability patients, the NNS was ~50 to identify patients with an SBO on CT.

## Introduction

Small bowel obstruction (SBO) is a common emergency department (ED) diagnosis, comprising up to 2% of ED patients with abdominal pain [1] and resulting in approximately 300,000 annual U.S. hospitalizations [2]. SBO occurs when there is a blockage or obstruction (such as from scar tissue adhesions or intestinal mass). If not diagnosed and treated within a timely manner, necrosis, perforation, or sepsis can occur. However, no specific set of clinical symptoms or physical signs are highly predictive of the diagnosis [3].

SBO is most commonly diagnosed via computed tomography (CT), the “gold standard” method. CT scans are rapid, non-invasive, and readily available in most EDs, either in the ED or nearby in the hospital. CT can identify an SBO transition point and reliably discover alternate pathology. Disadvantages of CT imaging for SBO include delays in care (time to CT performance and interpretation), radiation exposure, and, often, intravenous contrast exposure (which has been associated with allergies [4] and nephrotoxicity [5] among renally compromised patients). Radiation can be particularly harmful to vulnerable populations (e.g., children and fetuses). Additionally, in many hospitals, CT scans are not performed in the ED, necessitating the patient to leave the ED, which can be hazardous for a critically ill patient. Magnetic resonance imaging (MRI) can provide an accurate diagnosis of SBO without ionizing radiation but requires longer scanning time and produces inferior resolution [5]. It is also not readily available in most U.S. EDs, making it impractical [6]. 

Consequently, ED sonologist-performed point-of-care ultrasound (POCUS) or radiology-performed ultrasound is often preferred when certain diagnoses are under consideration due to limitations of CT and MRI when POCUS diagnostic accuracy is acceptable [7]. The diagnostic criteria for SBO on ultrasound (US) are similar to those with the other modalities: dilated loops of small bowel, free fluid (tanga sign), bowel wall thickening, and valvulae conniventes [8]. In addition, as a dynamic real-time modality, ultrasound can also visualize the absence of peristalsis. However, as POCUS is operator-dependent, there remains a risk of false positives and negatives, especially if the operator is not sufficiently experienced. This can lead to additional unnecessary testing (with associated risks and costs) [2,9]. Furthermore, the quality of the examination may be impacted depending on several patient characteristics, such as obesity, pregnancy, and presence of excessive intestinal gas [10]. 

Previous research suggests that POCUS has similar sensitivity (92%) and specificity (94%) compared to CT in diagnosing SBO [11]. POCUS can be performed at the bedside, avoids radiation, has immediate interpretation, and is less expensive [12]. Monte Carlo simulations performed with POCUS as the first diagnostic modality and CT scan as backup showed a cost savings of over $30 million and 143,000 fewer CTs performed annually in the United States [13].

Unlike other imaging modalities (in which the ordering and performing clinician are not the same), POCUS-performing/interpreting sonologists must recognize the risk of confirmation bias in the POCUS application [14]. Per Bayesian analysis [15], the likelihood of a diagnosis being true (post-test odds) following a diagnostic test is based on the ordering clinician’s pre-test probability and the test characteristics (sensitivity and specificity, from which positive and negative likelihood ratios can be calculated). Consequently, establishing pre-test probability is important in informing downstream diagnostic or therapeutic interventions, as pre-test probability influences post-test odds. Little research has been done on the role of POCUS sonologist’s pre-test probability and actual POCUS results in informing SBO diagnosis and management (e.g., modifying one’s plan to order a CT scan in cases with a low pre-POCUS probability of SBO) [16]. 

This study assessed the role of POCUS in SBO diagnosis, integrating pre-test probability and POCUS results to determine post-test odds and the number needed to scan (NNS) to modify diagnosis and management plans.

## Materials and methods

The Long Island Jewish Medical Center (LIJ), New Hyde Park, NY, is a 583-bed tertiary-care academic hospital serving a racially and socio-economically diverse population. The adult ED sees approximately 100,000 patients per year. 

Patients were included in the study if they fulfilled the following criteria: (1) presented between 2017 and 2023, (2) had signs or symptoms of SBO, (3) had a CT of the abdomen to evaluate for SBO, and (4) underwent a POCUS performed by one of 17 ED clinicians credentialed in performing and interpreting ultrasounds for SBO. Patients were approached for consent on a convenience basis, and the decision to perform POCUS for SBO was at the discretion of the performing ED clinician. The sonographer would be either the patient’s attending physician or physician assistant or an ED faculty member doing “scan shifts” and performing ultrasounds to facilitate ED care, but who otherwise did not have clinical responsibilities. Patients were excluded only if they were unwilling or unable to consent.

Research assistants recorded demographic data, past medical and surgical history, vital signs, and physical examination findings. The POCUS-performing clinicians provided pre-test probabilities for SBO: low (<20%), mild (<20-49%), medium (50-80%), or high (>80%). They then performed POCUS on the patient prior to CT imaging. The patient was placed in the supine position and adequately medicated for comfort. A linear (8-10 MHz) or curvilinear (4-6 MHz) probe was used based on the patient’s body habitus. A step-wise “mowing the lawn” technique was used across or up and down the patient’s abdomen. The probe was held in a transverse orientation. A checklist of sonographic findings was completed by the sonographer. Completion of the ultrasound took between 3 and 8 minutes. Each patient’s abdominal ultrasound was assessed for findings of SBO. 

SBO was defined as the presence of at least one of these criteria: (1) fluid-filled bowel with a diameter of >2.5 cm in three distinct bowel loops with adjacent collapsed bowel, (2) "to-and-fro" movement, (3) decreased or absent peristalsis, and (4) well-defined plicae circularis (“keyboard sign”).

POCUS results were compared with CT (the gold standard). Sensitivity, specificity, LR+, and LR− were calculated. 

Sample size

According to a study by Bujang and Adnan regarding minimum sample size for sensitivity and specificity analysis [17], assuming CT-confirmed SBO prevalence of 25% in this study and CT (gold standard) sensitivity of 100% for SBO, then, to detect a 10% difference (e.g., US has 90% sensitivity) with power of 80% and alpha (statistical significance) of 0.05, the estimated sample size of patients with an ultrasound-identified SBO is 30 patients. 

Descriptive statistics used were mean and percent. Student’s t-test was used to compare two percentages. Statistical significance was set a priori at p < 0.05.

The study was approved by the institutional review board.

## Results

One hundred six patients received POCUS examination; 17 POCUS-certified MDs and PAs performed scans (range, 1-24 patients). The mean patient age was 62.0 years (standard deviation, 17.2 years) (Table 1).

**Table 1:** Study patient characteristics

Characteristic	Number	%
Race		
Asian	16	15.1
Black	8	7.5
Hispanic	7	6.6
White	54	50.9
Other	21	19.8
Gender		
Female	60	56.6
Male	46	43.4

Twenty-four patients (23%) had a CT positive for SBO (Table 2); 92% of those were among patients in whom POCUS showed SBO.

**Table 2:** Number of POCUS and CT positive and negative for SBO CT, computed tomography; POCUS, point-of-care ultrasound; SBO, small bowel obstruction

	CT showed SBO	CT did not show SBO
POCUS showed SBO	24	8
POCUS did not show SBO	2	72

This yielded a POCUS sensitivity of 92% and specificity of 90% for SBO compared with a CT scan (Table 3).

**Table 3:** POCUS sensitivity, specificity, and positive and negative likelihood ratios CT, computed tomography; LR, likelihood ratio; POCUS, point-of-care ultrasound

	POCUS compared with CT
Sensitivity	92%
Specificity	90%
LR+	9.2
LR-	0.09

With a 23% prevalence of SBO among this cohort, the positive predictive value of a positive POCUS was 73%, and the negative predictive value of a negative POCUS was 97%. 

Sonologist pre-test probability was most strongly correlated with POCUS results (84%) and CT results (90.3%) when the pre-test probability was low-mild compared with high-moderate (Tables 4, 5). A positive POCUS among patients with a high-moderate pre-test probability was less likely than a negative POCUS among patients with a low-mild pre-test probability (p = 0.0003).

**Table 4:** Correlation between pre-test probability of SBO and POCUS findings POCUS, point-of-care ultrasound; SBO, small bowel obstruction

POCUS result	Pre-test probability	
	High (>80%) to moderate (50-80%)	Low (<20%) to mild (20-49%)
Positive POCUS	52%	16%
Negative POCUS	48%	84%

**Table 5:** Correlation between pre-test probability of SBO and POCUS findings CT, computed tomography; POCUS, point-of-care ultrasound; SBO, small bowel obstruction

CT result	Pre-test probability	
	High (>80%) to moderate (50-80%)	Low (<20%) to mild (20-49%)
SBO	43.2%	9.7%
No SBO	56.8%	90.3%

There was a greater correlation between POCUS and CT results among patients with a low-mild pre-test probability (p = 0.0062) than among those with a high-moderate pre-test probability (p = 0.4281) (Table 6).

**Table 6:** POCUS correlation with CT results by pre-test probability CT, computed tomography; POCUS, point-of-care ultrasound

	Pre-test probability		p-value
	High (>80%) to moderate (50-80%)	Low (<20%) to mild (20-49%)	
Correlation with CT when POCUS positive	74%	60%	0.4281
Correlation with CT when POCUS negative	86%	100%	0.0062
Correlation with CT overall (POCUS positive or negative)	80%	94%	0.0285

We performed a Bayesian analysis (Table 7) to determine post-test odds after integrating sonologist-assessed pre-test probability with POCUS results. Pre-test odds were calculated as pre-test probability/(1 − pre-test probability), and post-test odds were calculated as pre-test odds × LR [18]. For these analyses, we used the following values for pre-test probability:

(1) High probability (study range, >80%): we used the lower end of the range (81%) since it is unlikely that one can be so confident of a diagnosis of an internal organ disorder based on history and physical examination alone as to have substantially more than an 80% pre-test probability.

(2) Moderate (study range, 50-80%): we used the middle of the range (65%).

(3) Mild (study range, 20-49%): we used the middle of the range (34%).

(4) Low (study range, <20%): we used the upper end of the range (19%) to give the highest possibility of post-test odds, even in the event POCUS did not reveal an SBO.

**Table 7:** Post-test odds of SBO using Bayesian analysis POCUS, point-of-care ultrasound; SBO, small bowel obstruction

	Pre-test probability			
	High (81%)	Moderate (middle of range = 65%)	Mild (middle of range = 34%)	Low (19%)
POCUS positive for SBO	post-test odds = 39.6%	post-test odds = 17.5%	post-test odds = 4.8%	post-test odds = 2.1%
POCUS negative for SBO	post-test odds = 0.4%	post-test odds = 0.2%	post-test odds = 0.0%	post-test odds = 0.0%

Post-test odds allow calculation of NNS, defined as the number of POCUS examinations needed to be performed to benefit the patient or prevent an adverse procedure outcome. NNS is calculated as 100/absolute risk reduction% (e.g., 100/5% = NNS of 20) [19]. It is similar in concept to the number needed to treat (NNT) [20]. We determined the NNS for the scenarios in which POCUS was likely to change management: either order a CT scan when one was not initially inclined to do so or not order a CT scan when one was initially inclined to do so. When the pre-test probability of an SBO is high or moderate, then either a positive or a negative POCUS will likely result in a CT scan, either to confirm a POCUS-identified SBO or to investigate alternate causes (e.g., abdominal aortic aneurysm or intra-abdominal hemorrhage) of the patient’s presentation severe enough to prompt an initial high pre-test probability (e.g., significant pain, peritoneal signs, and markedly abnormal vital signs). Patients for whom POCUS will most likely affect diagnostic decisions for SBO are those with a low pre-test probability for SBO, who may or may not warrant a follow-up CT, depending on POCUS results. A positive POCUS, which occurs in 16% of low pre-test probability cases, would prompt a CT; ~2% of low pre-test probability patients (⅛ will have POCUS-positive low pre-test probability patients) will have SBO on follow-up CT (i.e., post-test odds, ~2%). Therefore, the NNS should be applied to circumstances of low pre-test probability, changing management from not ordering a CT to ordering a CT in 16% of cases. For providers whose practice is to place nasogastric tubes (NGT) based on POCUS results prior to a confirmatory CT, ~75% of patients with a high pre-test probability and a positive POCUS will have a CT positive for SBO. In such cases, the NNS (to make the decision to place an NGT) is 100/75% = ~1.3 (Table 8).

**Table 8:** Number needed to scan for low and high pre-test probability patients CT, computed tomography; NGT, nasogastric tube

	Pre-test probability	
	High (81%)	Low (19%)
Low pre-test probability (deciding order CT)		~6
High pre-test probability (deciding to place NGT pre-CT)	~1.3	

## Discussion

This study confirms prior studies’ findings of 90-95% sensitivity of POCUS for SBO compared with the gold standard (CT scan) [13,21]. Our findings of 92% sensitivity and 90% specificity yielded a positive likelihood ratio of 9.3 and a negative likelihood ratio of 0.09. POCUS is particularly good for “ruling out” SBO, with a 97% negative predictive value among all-comers (combining patients with high-moderate and those with low-mild pre-test probabilities); no patients with a low pre-test probability and a negative POCUS had SBO. Even with a high pre-test probability, the odds of an SBO in the presence of a negative ultrasound are 0.4%, well below the ~2% testing or admitting threshold other validated tools utilize. For example, the PERC Score for pulmonary embolism evaluation utilized a 1.8% false negative rate before reaching the threshold for ordering a CT scan [22]. Likewise, a low-probability Wells Score plus negative d-dimer yielded a low 1.3% false negative rate [23]. Finally, of patients with chest pain who were stratified using the HEART score, 1.7% had a near-term major adverse cardiac event, which was considered acceptable [24].

Sonologist pre-test probability plays a crucial role in interpreting ultrasound and CT findings. This is consistent with the findings of a study assessing whether emergency physicians could use POCUS in patients deemed clinically to have a moderate-high pre-test probability of appendicitis to diagnose acute appendicitis. Of 76 such patients, 36.8% were diagnosed with appendicitis, with a sensitivity of 42.8% and a specificity of 97.9% [25]. Another study assessing the safety and effectiveness of deep vein thrombosis (DVT) management techniques combining pre-test probability and d-dimer testing showed that pre-test probability aided in the diagnosis of DVT, with prevalence rates of proximal DVT of 4.5% in the low pre-test probability category, 18.8% in the moderate pre-test probability category, and 47.3% in the high pre-test probability category [26]. 

In sum, when sonologists have a low pre-test probability of SBO, an ultrasound that doesn’t demonstrate SBO effectively rules out SBO, with a negative predictive value of 99% if the prevalence of SBO among ED patients with abdominal pain is 2% [1]. If the ultrasound is positive for SBO, then post-test odds are 2.1%, and sonologists should use their judgment regarding progressing to CT, keeping in mind other commonly used validated tools that use a test threshold of ~2%. Among patients for whom there is a moderate-high pre-test probability for SBO, a positive POCUS should be followed up with a CT scan, as 74% of such patients had a CT-diagnosed SBO. Patients whose POCUS does not reveal an SBO should strongly be considered for a CT scan, as 10% of such patients had a CT-diagnosed SBO. NNS to modify one’s plan by ordering a CT for low pre-test probability patients is ~6; NNS to decide to place an NGT for high pre-test probability patients is ~1.3.

POCUS SBO, when performed by a trained ED sonologist, would best be used as a screening tool for SBO. When a low probability of SBO is found on POCUS, other diagnoses should be entertained. If there is a high probability of SBO on POCUS, it should be followed with a CT scan to further evaluate and determine surgical planning.

This study has several limitations. First, it was a single-site study. Second, subjects were not selected randomly; instead, they were selected on a convenience basis, and it was at the clinician’s discretion whether to perform a POCUS or order a CT scan. Additionally, not all ED clinicians are POCUS-certified; the study relied on clinicians being aware of the study and either performing a POCUS or asking a POCUS-certified clinician to do so, which was limited by the certified sonologists’ availability. Sonologists may have chosen patients on whom to perform POCUS based on convenience, teaching ultrasound opportunity, ability to consent, or pre-test suspicion. Finally, many EDs do not have ultrasound-trained physicians available, limiting this study’s generalizability.

## Conclusions

Utilizing Bayesian analysis can help determine the appropriate use and interpretation of POCUS in the diagnosis of SBO, as well as establish the NNS to affect change in management, typically referring to the decision to order an abdominal CT. POCUS offers valuable support to clinicians and patients in diagnosing SBO, primarily in ruling out SBO (i.e., among patients with a low pre-test probability, and negative POCUS excludes SBO and obviates the value of CT for this diagnosis). NNS to modify one’s plan by ordering a CT for low pre-test probability patients is ~6; NNS to decide to place an NGT for high pre-test probability patients is ~1.3. Among patients with a high-moderate pre-test probability, POCUS probably does not affect the decision to order a CT, as such patients will likely undergo a CT to find either an SBO or alternate diagnosis. However, POCUS can identify important etiologies that might alter care, such as AAA or free fluid in the abdomen indicative of hemorrhage.
